# Autonomy supportive environments and mastery as basic factors to motivate physical activity in children: a controlled laboratory study

**DOI:** 10.1186/1479-5868-9-16

**Published:** 2012-02-21

**Authors:** James N Roemmich, Maya J Lambiase MS, Thomas F McCarthy, Denise M Feda, Karl F Kozlowski

**Affiliations:** 1 Department of Pediatrics, School of Medicine and Biomedical Sciences, University at Buffalo, Buffalo, New York 14214-3000, USA; 2 Department of Exercise and Nutrition Sciences, School of Public Health and Health Professions, University at Buffalo, Buffalo, New York 14214-3000, USA

## Abstract

**Background:**

Choice promotes the experience of autonomy, which enhances intrinsic motivation. Providing a greater choice of traditional active toys may increase children's activity time. Mastery also increases intrinsic motivation and is designed into exergames, which may increase play time of a single exergame, reducing the need for choice to motivate activity compared to traditional active toys. Providing both choice and mastery could be most efficacious at increasing activity time. The energy expenditure (EE) of an active play session is dependent on the duration of play and the rate of EE during play. The rate of EE of exergames and the same game played in traditional fashion is not known. The purpose was to test the basic parameters of choice and mastery on children's physical activity time, activity intensity, and energy expenditure.

**Methods:**

44 children were assigned to low (1 toy) or high (3 toys) choice groups. Children completed 60 min sessions with access to traditional active toys on one visit and exergame versions of the same active toys on another visit.

**Results:**

Choice had a greater effect on increasing girls' (146%) than boys' (23%) activity time and on girls' (230%) than boys' (minus 24%) activity intensity. When provided choice, girls' activity time and intensity were no longer lower than boys' activity time and intensity. The combination of choice and mastery by providing access to 3 exergames produced greater increases in physical activity time (1 toy 22.5 min, 3 toys 41.4 min) than choice alone via access to 3 traditional games (1 toy 13.6 min, 3 toys 19.5 min). Energy expenditure was 83% greater when engaging in traditional games than exergames.

**Conclusions:**

Boys and girls differ in their behavioral responses to autonomy supportive environments. By providing girls with greater autonomy they can be motivated to engage in physical activity equal to boys. An environment that provides both autonomy and mastery is most efficacious at increasing physical activity time. Though children play exergames 87% longer than traditional games, the rate of energy expenditure is 83% lower for exergames than traditional indoor versions of the same games.

## Background

Engaging in adequate physical activity is considered an essential component for the maintenance of weight loss and provides many other health benefits for children [1]. However, many children do not engage in adequate physical activity to receive all of its health benefits [2]. Unfortunately, treatment programs based on a number of behavioural change models and applied in a number of settings have not demonstrated efficacy for promoting sustained increases in youth physical activity after cessation of the program [3]. The development of more effective behavioural interventions for increasing youth physical activity depends on improving our understanding of basic factors that influence human physical activity behaviour, and then translating that knowledge into interventions.

One potentially important basic factor to motivate greater physical activity behaviour is choice. The association between choice and intrinsic motivation to engage in a behaviour is a central theme of Self-Determination Theory (SDT). Constructing the environment so that it provides opportunities to make choices allows the individual to experience autonomy, which enhances intrinsic motivation to engage in a behaviour [4,5]. Several studies have established relationships between autonomy and physical activity behaviour [6-8]. Investigators have recently begun to base physical activity interventions on SDT with initial results of improved autonomous self-regulation of physical activity and greater participation in physical activity [9].

If autonomy does increase intrinsic motivation to engage in physical activity, then presenting children with a choice of options for physically active play that differ in characteristics such as the equipment used, necessary movement patterns and skills, or strategy should increase physical activity participation. There are few controlled laboratory studies of the effect of choice on children's physical activity participation. A recent study manipulated access to the number of resistance training machines to study the effect of choice on physical activity participation [10]. Children performed a greater number of repetitions and lifted more total weight when provided access to 7 resistance training machines than when provided access to only 1 machine. While this model allowed choice to be easily manipulated while controlling for the mode of exercise, the results of this study are limited to resistance training.

The current study extends previous research by providing children access to more traditional and commonly used physically active games. It also extends previous research by determining whether increasing the choice of exergames, the genre of videogames that combine gaming and exercise, has similar effects on increasing physical activity as traditional active toys. Exergames have become a part of youth culture so it is best to understand how they can be used to motivate healthy behaviours. SDT has been used as a framework when designing traditional computer games intended to improve learning [11,12] and promote health behaviour change [13] through increased intrinsic motivation. A greater sense of autonomy predicts motivation to play sedentary computer games as measured by the preference for continued play of the game also increases enjoyment of the game [14]. To the best of our knowledge, the influence of autonomy on exergame play has not yet been tested. There is also little information on the influence of autonomy on the play of traditional active games, though previous research has demonstrated that an intervention based in part on SDT and promoting autonomy of activity choices increased in total physical activity in underserved adolescents [15].

Mastery is a psychological force that motivates a child to persist at developing proficiency for completing a task. Mastery is specifically designed into many exergames, in that initial levels of the game must be mastered before being allowed to advance to the next level. A motivational climate that focuses on mastery rather than competition increases intrinsic motivation for physically active pursuits [16-19]. By increasing intrinsic motivation to persist at a given task, mastery may promote increased adherence to a single exergame. This may reduce the need for choice to promote greater physical activity, resulting in greater physical activity when no choice is available. However, when both choice and a mastery-focused motivational environment are provided, such a combination could be most efficacious at increasing intrinsic motivation and physical activity time.

To best compare the ability of choice and mastery to motivate physical activity behaviour of traditional games and exergames, key study design issues include matching both types of games for activity mode and choosing traditional games designed for indoor play, particularly play inside a home. By doing so, differences in activity amount and intensity can be attributed to inherent differences in game design.

Exergames may compete with, or be used as a substitute for, traditional active play. Given the importance of physical activity for the maintenance of a healthy body weight, it is vital to understand the energy expenditure associated with exergames and traditional active indoor games that are played at home. There are necessary motor movements to engage in play with traditional active toys including the need to handle, chase and retrieve balls, hockey pucks or other equipment. Conversely, play with exergames is dependent on handheld controllers that are programmed to mimic traditional game play and the software resets the play environment while the participant rests. These differences inherent to game type, along with potential differences in the intensity and duration of play likely produce differences in energy expenditure of traditional and exergames. Several studies have compared the energy expenditure of exergames to sedentary videogames or other modes of activity such as treadmill walking [20-28]. While exergames increase energy expenditure above sedentary levels, the energy expenditure during exergames has not yet been compared to traditional home versions of the same activities. Thus, the purpose was to determine whether the basic factors of choice and mastery promote an increase in physical activity time and intensity. A second purpose was to compare children's rate of energy expenditure while playing exergames and indoor home versions of the actual traditional games.

## Methods

### Subjects

Study participants included 22 boys (20 White, 2 Black) and 22 girls (19 White, 1 Black, 2 Asian) between the ages of 8-12 years, with a BMI lower than 95th percentile for age and sex (Table 1). Children were recruited from a database of over 10,000 families that had called the laboratory to receive information about previous studies and had given consent to be contacted for future studies. Only those families with children who appeared to meet entry criteria based on information in the database were called. Great care was taken to ensure that children had not participated in a previous study that would cause real or perceived influence on the outcome measures. All children recruited indeed met entry criteria and were enrolled in the study. All children completed all study dates and all measurements. A subsample of these children (6 boys, 6 girls) was recruited to complete ergospirometry measures of the metabolic cost of exergames and indoor versions of the same games in a traditional format. The subsample was selected based on the children's ability to follow direction and complete the measurements during the main study and; through interview, their willingness to wear a backpack and face mask for the ergospirometry measures. Again, all children approached for the substudy chose to participate and completed all of the measurements. There were no significant differences in the age (*p *≥ 0.85), height (*p *≥ 0.92), weight (*p *≥ 0.48), body mass index (BMI) percentile (*p *≥ 0.41), or socioeconomic status (SES, *p *≥ 0.92) of the boys and girls in the whole sample and subsample. Exclusion criteria included any disorders that would affect the ability to exercise, including cardiovascular, neuromotor, cognitive or orthopedic disorders or severe or uncontrolled asthma. The research was carried out in compliance with the Helsinki Declaration. Parents provided written informed consent for their child to participate and each child gave their written assent to participate. Separate written informed consent and assent were obtained for participation in the study to determine the metabolic costs of traditional and exergames. The University at Buffalo Children and Youth Institutional Review Board approved this study.

**Table 1:** Physical characteristics, demographics and activity attitudes

	Boys		Girls	
	
	No choice n = 11	Choice n = 11	No choice n = 11	Choice n = 11
Age (yr)	10.6 ± 0.9	10.2 ± 1.4	9.6 ± 1.6	10.2 ± 1.3
Height (cm)	145.1 ± 14.4	146.1 ± 14.4	138.6 ± 11.7	142.6 ± 6.8
Weight (kg)	41.0 ± 3.1	39.0 ± 12.6	34.4 ± 8.8	36.4 ± 6.7
BMI %ile	74.8 ± 17.2	51.3 ± 32.1	54.2 ± 24.9	50.8 ± 25.7
SES	50.9 ± 10.5	56.8 ± 7.3	49.8 ± 7.7	49.2 ± 11.5
Wii play (hr/wk)	4.0 ± 1.4	2.0 ± 1.9	2.0 ± 2.7	2.7 ± 3.9
Physical activity attitudes				
Adequacy	23.8 ± 3.4	21.8 ± 5.1	21.6 ± 3.3	21.8 ± 5.7
Predilection	30.5 ± 3.9	27.2 ± 5.7	30.9 ± 4.0	29.2 ± 5.3
Enjoyment^a^	11.5 ± 0.7	10.5 ± 2.7	9.6 ± 2.0	9.2 ± 2.9

Data are mean ± SD

^a^*p *< 0.05 for main effect of gender

There were no significant main effects of choice group

*SES *socioeconomic status. An SES of 40 through 50 is equivalent to medium size business owners, minor professionals and technical jobs, such as computer programmers, real estate agents, sales managers, social workers and teachers.

Physical activity attitude scales - scored such that higher scores reflect greater perceived self-efficacy. The range is 19 to76 when summing the component scale scores to yield a total score.

### Research procedures

#### Choice and physical activity experiment

Children and a parent reported to the laboratory for 3 visits. Height and weight of the child were measured at the initial visit. Children then sampled each of the traditional physically active games (mini basketball, punching bag and gloves, mini floor golf, mini floor hockey) and their exergame counterparts (Wii Sports Resort Basketball, Wii Sports Boxing, Wii Sports Golf, Mario & Sonic's Winter Olympic Games Hockey) for 3 min. The traditional games were carefully chosen in that they were designed to be conducted indoors and within the same sized room as an exergame. Children played both versions of the games in a 3.1 m by 4.0 m experimental room. Children first sampled all of the traditional toys or all of the exergames as categories with the order of sampling of the categories counterbalanced across subjects. After sampling all 10 toys, children rank ordered their liking of each paired traditional game and matched exergame (1 being the most liked pair and 5 being the least liked pair). Access to specific toys during the experiment was determined by the rank scores for the paired traditional toy and its exergame counterpart. A parent completed a standardized demographic questionnaire to assess socioeconomic level [29], family income, parent educational level and racial/ethnic background.

Boys and girls were equally and randomly assigned to no choice (1 toy) or choice (3 toys) groups. During visits 2 and 3, the children completed two 60 min sessions with access to either 1 or 3 traditional toys at one visit and the same number of exergames at another visit. The order of access to traditional toys and exergames was counterbalanced across subjects. Children in the 1 toy (no-choice) group had access to only their most highly ranked traditional active toy or its exergame counterpart. Children in the 3 toy group had access to their 3 most highly ranked traditional toys or their exergame counterparts. For the 3 toy group, each exergame was loaded on one of 3 game counsels so that children did not have to wait to remove, reload, and set-up an exergame when switching from one exergame to another. Both groups of children had equal access to alternative sedentary behaviours of resting quietly, reading children's magazines, and puzzles during both of the choice sessions. Only the traditional active toys or exergames that the children were assigned to play with were in the laboratory room, along with the sedentary alternatives. Children wore an accelerometer (GT1M, ActiGraph, Pensacola, Florida) to monitor their physical activity intensity. No constraints were placed on the intensity or amount of physical activity that the children needed to engage in. Children were studied separately, without the presence of their parent, given identical instructions, and no verbal encouragement from the investigators to eliminate the influence of peers, parents, and investigators on physical activity/sedentary choices.

#### Metabolic cost of physical activity experiment

Children completing the ergospirometry measures reported to the laboratory for 1 additional visit. Children were fitted with a Polar heart rate monitor (Polar Electro Inc., Lake Success, NY), Actigraph accelerometer (Pensacola, Florida), and an Oxycon portable ergospirometry system (Yorba Linda, CA) and then rested quietly for 15 min. Children then performed each of the sedentary activities, and played with each of the traditional physically active toys and exergames for 4 min each. No information was provided to the children regarding the intensity of physical activity. The physiologic measurements began with activities of lowest metabolic cost and finished with those of greatest metabolic cost. This prevented a previous higher energy expenditure activity contaminating data of later lower-energy expenditure activity. The mask was removed after each activity and the child rested for 3 min before beginning the next activity.

### Measurements

#### Anthropometry

Weight was measured to the nearest 0.1 kg using an electronic weight scale and height to the nearest 0.01 cm using a calibrated stadiometer. BMI (kg/m^2^) percentile was calculated in relationship to 50th BMI percentile for children based on their sex and age [30].

#### Liking of activities

Liking of each active toy was determined using a 10 point Likert scale anchored by 'do not like it at all' on the left and 'like it very much' on the right. Likert scores are a reliable and valid method to assess liking or hedonics as an affective rating of a behaviour [31] and are positively correlated with physical activity participation [32].

#### Accelerometry

Children wore an ActiGraph GT1M accelerometer around their waist to determine the amount of physical activity during the experimental session. Prior to each testing session, the accelerometer unit was initialized for a 60-s epoch and attached to an elastic belt that was worn around the waist. The ActiGraph unit was seated on the right mid-axillary line at the level of the iliac crest. The ActiGraph GT1M is a valid and reliable method of estimating physical activity in children [33]. Time spent in moderate-to-vigorous physical activity (MVPA) was determined using a validated cut-point for youth age 8 to 12 years [33]. The average counts/minute also served as an outcome variable for the choice experiment; and as the outcome variable for the metabolic cost of physical activities experiment, which had short trials of only 4 min.

#### Total time engaged in active play

The total time children played with the active games or engaged in one of the sedentary pursuits was measured by observing the child during the experimental session from an adjacent control room. Time playing with active games was defined as the duration that the children were engaged with one of the active choices, regardless of the intensity of the activity. Sedentary time was accrued when the child was engaged in one of the alternative sedentary behaviours of resting quietly, reading children's magazines, and puzzles. Children could engage in only one activity at a time.

#### Wireless portable ergospirometry and heart rate

The rate of oxygen consumption and heart rate during rest and during each activity were measured using the Oxycon Mobile (Yorba Linda, CA), which is a portable (950 g) indirect calorimetry system. The portable system was slipped over the child's shoulders and held in place by a vest and harness. An appropriately sized face mask (Hans Rudolph, Kansas City, MO) was placed snugly over the child's nose and mouth and held in place by a harness. The mask was fitted with a low-resistance bidirectional flow sensor unit that measured the volume of inspired and expired air. A sample line connecting the flow sensor unit to the analyzer box delivered expired air for the determination of the concentration of O_2 _and CO_2_. After a 30 min warm-up, and immediately before data collection of each subject, the flow sensors and gas analyzers were calibrated following the manufacturers recommendations. Heart rate was measured by a wireless coded transmitter worn around the chest. The mean gas exchange variables and heart rate recorded between minute 2 and 4 of each activity were used as data. Energy expenditure (kcal^.^min^-1^) was calculated from the gas exchange data [34].

#### Physical activity attitudes

The Children's Self-Perceptions of Adequacy in and Predilection of Physical Activity scale [35] was used to measure children's attitudes towards physical activity. The scale measures 3 factors; perceived adequacy (confidence in), predilection (preference for), and enjoyment of physical activity. The scale has a test-retest validity of 0.70 to 0.91 for school grade groups of 4th to 8th grade [35]. The total scale score is correlated with usual physical activity (*r *between 0.59 and 0.76) and motor coordination (*r *between 0.70 and 0.82) [35].

### Analytic plan

Differences in physical characteristics, demographics, liking of the sedentary alternatives, and physical activity attitudes were tested with separate two-way ANOVA with gender (boy, girl) and choice group [single toy (no choice), 3 toys (choice)] as between factors. Differences in liking of activity choices, minutes engaged in physical activity and physical activity counts/minute were tested with separate three-way ANOVA models with gender and choice group as between factors and toy design (traditional/exergame) as a within subjects variable. Univariate correlations were used to test the association between physical activity attitudes and physical activity.

For the metabolic cost of physical activity experiment, differences in heart rate, physical activity counts/min, and the rate of energy expenditure were determined with separate ANOVA with gender as a between factor and toy design (traditional/exergame) and mode of activity (reading/puzzles/golf/hockey/basketball/boxing) as within subjects variables. Significant interactions were explored with contrasts. Effect sizes were calculated using the generalized eta square (h^2 ^generalized) statistic, which is preferred over h^2 ^and partial h^2 ^for analysis of variance models that include repeated measures [36,37]. h^2 ^generalized effect sizes are interpreted as small 0.02, medium 0.13, and large 0.26 [36].

## Results

### Physical characteristics and demographics

As shown in Table 1, there were no gender F(1, 40) ≥ 3.22, *p *≥ 0.09 or choice group F(1, 40) ≥ 3.07, *p *≥ 0.09 differences in physical characteristics, hours/week playing exergames, liking of the sedentary alternatives, physical activity adequacy, or physical activity predilection. Girls; however, had a lower F(1, 40) = 4.97, *p *< 0.05 physical activity enjoyment than boys. As shown in Table 2, for liking of the physically active games, there was one significant gender effect in that boys liked hockey more F(1, 40) = 9.14, *p *< 0.004 than girls. There were two differences in liking due to toy design. Children had a greater F(1, 40) = 17.76, *p *< 0.001 liking of traditional mini indoor basketball than the exergame version of basketball and a greater F(1, 40) = 10.81, p < 0.002 liking of the exergame version of golf than indoor mini golf. There were no significant F(1, 40) ≥ 1.85, *p *≥ 0.18 choice group effects for liking of the physically active games.

**Table 2:** Liking of the sedentary alternatives and of the traditional active toys and exergames

	Boys		Girls	
	Traditional	Exergame	Traditional	Exergame
Magazines	7 ± 1		7 ± 1	
Puzzles	6 ± 1		5 ± 1	
Basketball^a^	9 ± 1	7 ± 1	8 ± 1	7 ± 1
Boxing	7 ± 1	8 ± 1	8 ± 1	8 ± 1
Golf^a^	6 ± 1	7 ± 1	6 ± 1	8 ± 1
Hockey^b^	8 ± 1	8 ± 1	6 ± 1	6 ± 1

Data are mean ± SE

^a ^*p *< 0.05 for main effect of toy design

^b ^*p *< 0.05 for main effect of gender

Liking measured using a 1 to10 point Likert scale anchored by 'do not like it at all' on the left (score of 1) and 'like it very much' on the right (score of 10).

### Choice and physical activity time

**Figure 1:**
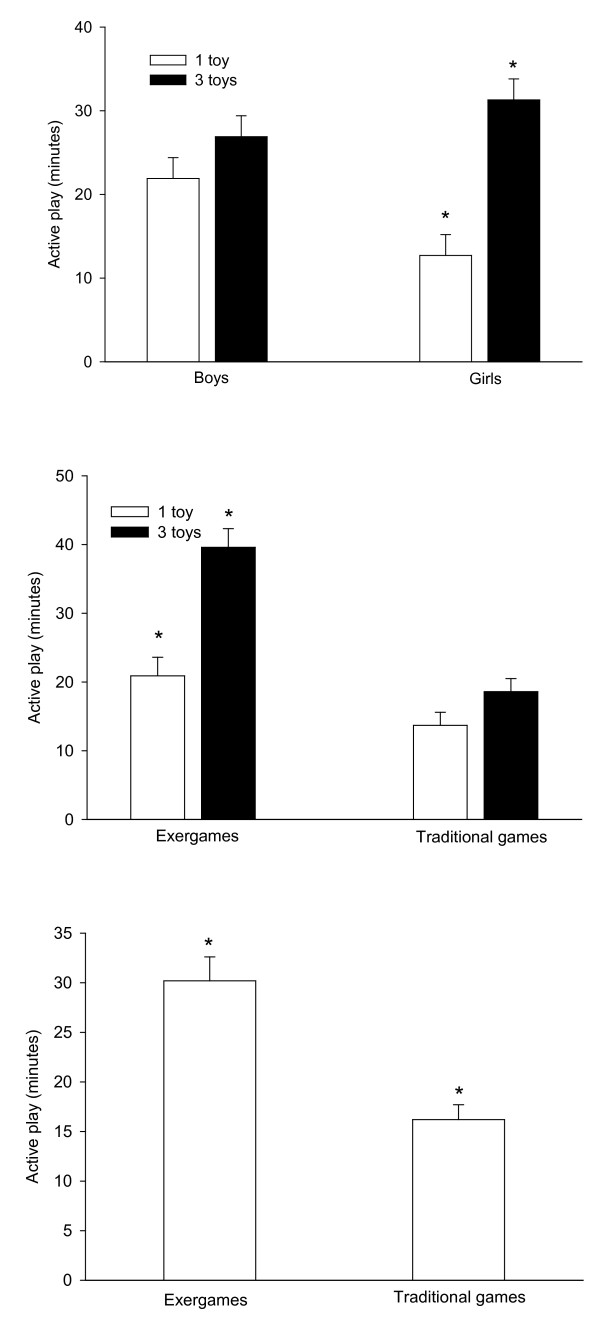
**Active play time during the 60 min free-choice session**. Top panel: minutes of active play of boys and girls assigned to the no choice (1 active toy) and choice (access to 3 active toys) groups. *Indicates a significantly (*p *< 0.05) greater physical activity time of the girls assigned to the 3 toys group compared to the 1 toy group. Middle panel: minutes of active play of children in the no choice (1 active toy) and choice (access to 3 active toys) groups who completed two 60 min sessions with access to either 1 or 3 traditional toys at one visit and the same number of exergames at another visit. *Indicates a significant (*p *< 0.05) greater time playing exergames of children assigned to the 3 toy group compared to the 1 toy group. Bottom panel: minutes of active play of children while playing with active traditional toys and exergames. *Indicates a significant (*p *< 0.05) greater minutes of activity lay with exergames compared to traditional indoor games.

As shown in the top panel of Figure 1, providing choice increased time spent being physically active F(1, 40) = 21.58, *p *< 0.001, h^2 ^generalized = 0.41 and had a greater effect (gender by choice group interaction: F(1, 40) = 7.16, *p *< 0.02, h^2 ^generalized = 0.18) on increasing girls' (146% increase, F(1, 40) = 26.80, *p *< 0.001) than boys' (23% increase, F(1, 40) = 1.94, *p *≥ 0.17) physical activity. As shown in the middle panel of Figure 1, there was a significant F(1, 40) = 10.07, *p *< 0.003, h^2 ^generalized = 0.17 interaction of choice group by toy design for physical activity time as choice increased time engaged in exergames (90% increase, F(1, 40) = 23.33, *p *< 0.001) more than traditional games (36% increase, F(1, 40) = 3.36, *p *≥ 0.07). There was also a significant main effect of toy design (bottom panel, Figure 1) in that children spent 87% more time in active play (F(1, 40) = 42.57, *p *< 0.001) when given access to exergames than traditional games.

### Choice and activity intensity

**Figure 2:**
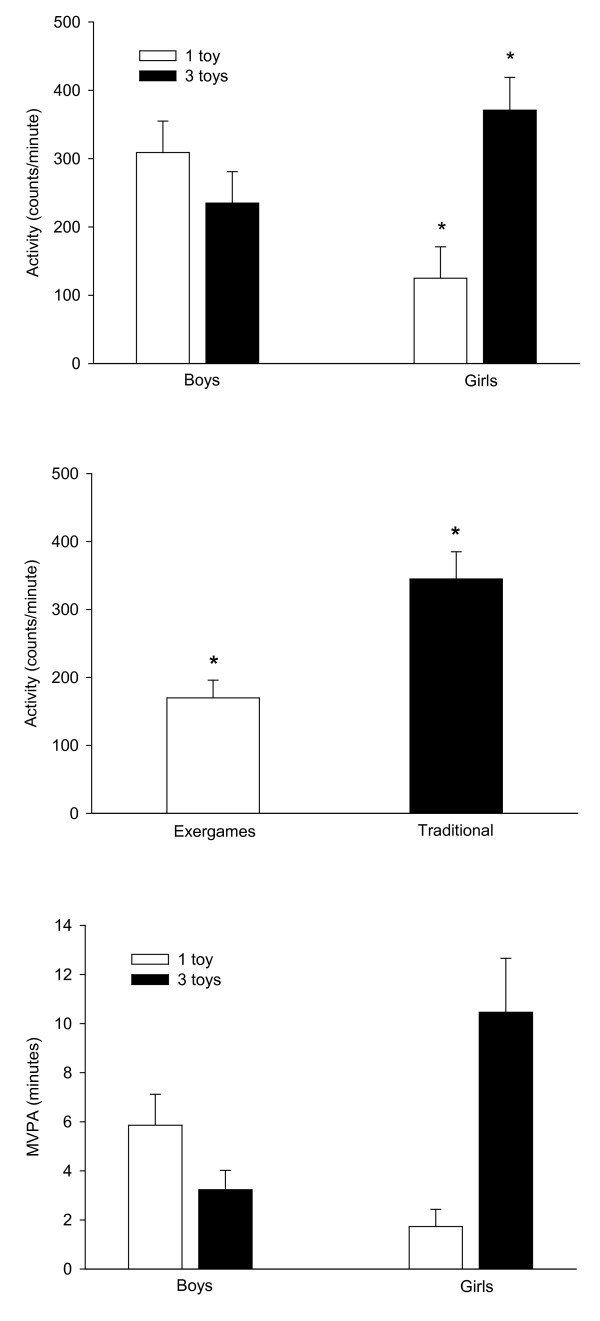
**Average activity intensity of the 60 min free-choice session**. Top panel: average rate of activity counts (counts/minute) of boys and girls assigned to the no choice (1 active toy) and choice (access to 3 active toys) groups. *Indicates a significantly (*p *< 0.05) greater activity intensity of the girls assigned to the 3 toys group compared to the 1 toy group. Middle panel: activity intensity of children while playing with active traditional toys and exergames. *Indicates a significant (*p *< 0.05) greater activity intensity while playing traditional indoor games compared to exergames. Bottom panel: moderate-to-vigorous physical activity (MVPA, minutes) of boys and girls assigned to the no choice (1 active toy) and choice (access to 3 active toys) groups. *Indicates significantly (*p *< 0.05) greater MVPA of the girls assigned to the 3 toys group compared to the 1 toy group.

As shown in the top panel of Figure 2, the average activity intensity across the entire 60 min session, as measured by accelerometry, was dependent on the interaction of gender and choice group F(1, 40) = 13.03, *p *< 0.001, h^2 ^generalized = 0.45. Increasing choice from 1 to 3 toys increased girls' activity counts/min by 230% (F(1, 40) = 16.45, *p *< 0.001) while it decreased, but did not significantly change, boys' activity counts/min by 24% (F(1, 40) = 1.10, *p *≥ 0.30). There was a significant main effect of toy design F(1, 40) = 20.73, *p *< 0.001, h^2 ^generalized = 0.39 as the average activity intensity was 107% greater when children had access to traditional active games than exergames (Figure 2, middle panel). As shown in the bottom panel of Figure 2, MVPA was dependent on the interaction of gender and choice group F(1, 40) = 17.25, *p *< 0.001, h^2 ^generalized = 0.38. Increasing choice from 1 to 3 toys increased girls' MVPA 505% (F(1, 40) = 20.34, *p *< 0.001) while it decreased boys' MVPA by 82% (F(1, 40) = 1.86, *p *≥ 0.18). There was also a significant F(1, 40) = 12.87, *p *< 0.001, h^2 ^generalized = 0.20 main effect of toy design on MVPA with children choosing to participate in 142% greater MVPA when playing with traditional active toys (7.5 ± 1.2 min) than exergames (3.1 ± 0.9 min).

### Univariate correlations of physical activity attitudes and physical activity

Girls' perceived adequacy of physical activity was correlated with their accelerometer counts/min during both the traditional (r = 0.46, *p *< 0.05) and exergame (r = 0.52, *p *< 0.05) sessions. Girls enjoyment of physical activity was correlated with their accelerometer counts/min during the traditional (r = 0.46, *p *< 0.05), but not the exergame (r = 0.18, *p *≥ 0.42) sessions. Physical activity attitudes were not significantly (*p *≥ 0.70) correlated with boys' physical activity.

### Metabolic cost of traditional games and exergames

**Figure 3:**
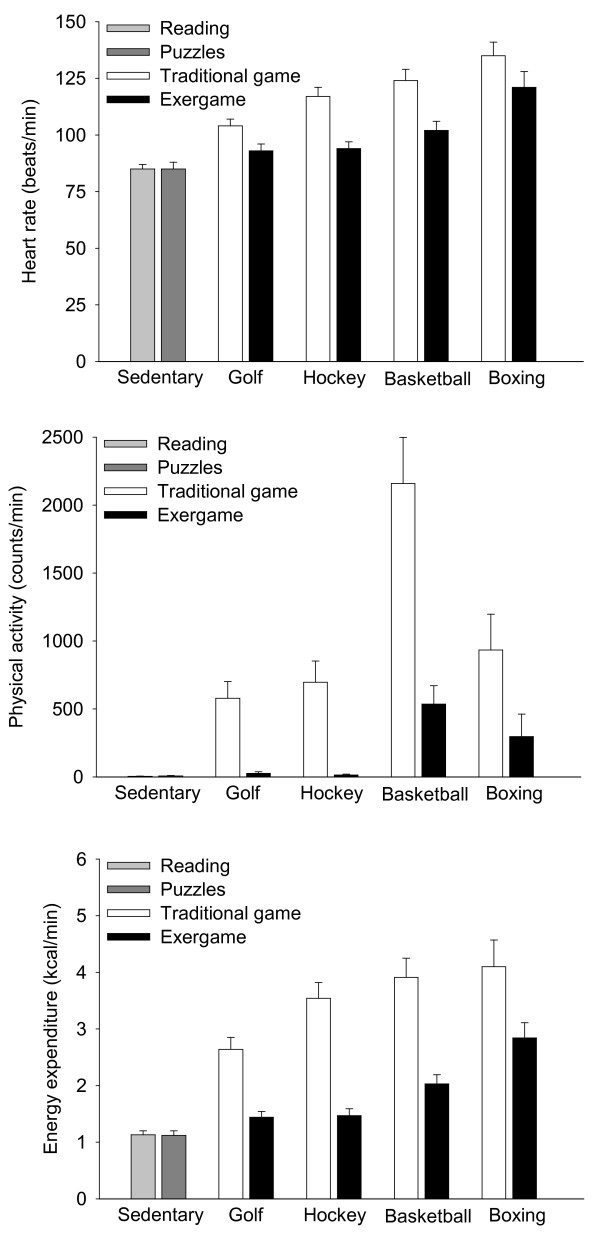
**Average activity intensity of the 60 min free-choice session**. Heart rate (top panel), rate of activity counts (middle panel), and rate of energy expenditure (bottom panel) while the children were engaged in sedentary behaviours (reading, puzzles) and while engaged in traditional and exergame versions of golf, hockey, basketball, and boxing. See Results section for a summary of the statistical differences.

Average heart rate (top panel), accelerometer counts/min (middle panel), and energy expenditure (kcal/min, bottom panel) while engaged in the sedentary activities and each of the exergames and corresponding traditional active games are shown in Figure 3. For exergames, there were significant main effects of mode of activity for heart rate F(1, 10) = 38.95, *p *< 0.001, h^2 ^generalized = 0.46 and for the rate of energy expenditure F(1, 10) = 35.94, *p *< 0.001, h^2 ^generalized = 0.57. Post-hoc analyses revealed that both heart rate F(1, 10) = 19.8, *p *< 0.001 and the rate of energy expenditure F(1, 10) = 7.94, *p *< 0.01 were greater during each mode of activity than during rest (reading or puzzles). There was also a significant main effect of mode of activity F(1, 10) = 15.95, *p *< 0.001, h^2 ^generalized = 0.41 for accelerometer counts/min. Post-hoc analyses determined that counts/min were greater F(1, 10) = 20.16, *p *< 0.001 than rest for the exergame version of basketball and marginally greater F(1, 10) = 3.17, *p *≥ 0.09 than rest for the exergame version of boxing. For traditional versions of the activities there were significant main effects of mode of activity for heart rate F(1, 10) = 98.38, *p *< 0.001, h^2 ^generalized = 0.75, counts/min F(1, 10) = 22.50, *p *< 0.001, h^2 ^generalized = 0.58, and the rate of energy expenditure F(1, 10) = 45.25, *p *< 0.001, h^2 ^generalized = 0.63. Post-hoc analyses showed that each of the traditional modes of activity were greater than rest for heart rate F(1, 10) = 58.86, *p *< 0.001, counts/min F(2, 10) = 7.44, *p *< 0.01, and the rate of energy expenditure F(1, 10) = 25.47, *p *< 0.001.

The interaction of toy design by mode of activity was significant F(1, 10) = 5.71, *p *< 0.004, h^2 ^generalized = 0.05 for heart rate. The average heart rate while playing each of the traditional games was greater F(1, 10) = 7.37, *p *< 0.02 than during each of the corresponding exergames. The difference in heart rate between the exergame version of golf and its traditional counterpart was smaller F(1, 10) = 9.24, *p *< 0.006 than the differences in heart rate between the exergame and traditional indoor versions of hockey and basketball.

There was a significant interaction F(1, 10) = 3.98, *p *< 0.02, h^2 ^generalized = 0.12 of toy design by mode of activity for physical activity counts/min. The counts/min while playing each of the traditional games was greater F(1, 10) = 4.13, *p *< 0.05 than during each of the corresponding exergames, with the greatest difference F(1, 10) = 5.12, *p *< 0.03 occurring during basketball.

The interaction of toy design by mode of activity was significant F(1, 10) = 4.37, *p *< 0.02, h^2 ^generalized = 0.03 for the rate of energy expenditure. The rate of energy expenditure while playing each of the traditional games was greater F(1, 10) = 5.23, *p *< 0.03 than during each of the corresponding exergames. The difference in rate of energy expenditure between the exergame version of golf and its traditional counterpart was smaller than the differences between the exergame and traditional indoor versions of hockey F(1, 10) = 7.61, *p *< 0.01 and basketball F(1, 10) = 6.14, *p *< 0.02. There were no significant (*p *≥ 0.22) main effects of gender, no significant (*p *≥ 0.13) interactions of gender with toy design or mode of activity, or any significant interactions of gender by toy design by activity mode (*p *≥ 0.08) for any of the outcomes.

## Discussion

The current study tested the basic factor of increasing choice on youth physical activity. Given that past research has demonstrated the importance of motivation for children to engage in physical activity [32,38], the current study relied on SDT as a framework to understand how choice can increase intrinsic motivation to engage in physical activity. According to SDT, setting the environment so that it provides opportunities to make choices allows the individual to experience autonomy, which enhances intrinsic motivation to engage in a behaviour at that time and in the future [4,5]. Providing autonomy through an increased choice of active toys in a child's environment increased the duration and intensity of physical activity, especially of girls. When presented with no choice, girls were neither as active as long nor as intense as boys. This is not surprising as girls are typically less active than boys [39,40], but by simply providing girls with a greater choice of active toys they can be motivated to engage in physical activity equal to boys.

There are few controlled laboratory studies of the effect of choice on children's physical activity participation. We manipulated access to the number of resistance training machines [10]. This model allowed choice to be studied while controlling for exercise mode. Children performed greater repetitions and lifted more total weight when provided access to 7 resistance training machines than when provided access to only 1 machine.

More recently, we (unpublished results) provided children access to no choice (1 toy) or two different levels of choice (3 toys or 5 toys) of traditional physically active games such as indoor basketball, hockey, and jumping games. Consistent with the current results, boys engaged in 130% longer active play and 150% greater activity intensity than the girls in the no choice (1-toy) group. However, providing a choice of 3 or 5 active toys increased physical activity time (42% versus 190%) and intensity (8% versus 180%) more for girls than boys. The consistency of these results with those of the current study argues for a gender difference in children's responsiveness to choice. Qualitative research agrees with these quantitative results in that twice as many girls than boys identify having a choice of activities as a motivational factor for physical activity [41].

Yet, SDT would suggest that the need for autonomy to promote motivation is an equally relevant and important need for both genders [42]. SDT has been applied to both boys and girls in physical activity and physical education settings [6-8]. However, there is growing evidence that boys and girls can differ in their interpretations of, and behavioral responses to, the same motivational events and autonomy supportive environments [8,43]. Despite participating in the same physical education lessons designed to provide autonomy-supportive strategies, girls reported receiving more optimal levels of challenge, autonomy-support and enjoyment; while boys reported greater perceived competence [8].

Such gender differences in motivational experiences and physical activity responses to autonomy-supportive environmental manipulations may be due to underlying gender differences in usual physical activity participation and previous experiences that have influenced attitudes towards physical activity and decisions to engage in physical activity when encountering such environments. One reason for this gender difference may be baseline physical activity levels. Boys' physical activity under a no choice condition is 30% to 200% greater than girls. Likewise, during periods of unorganized free-play on playgrounds, girls engage in less total and moderate-to-vigorous physical activity than boys [39,40]. The law of initial values would argue that the greater baseline physical activity of boys would reduce the tendency for their physical activity to increase when provided greater autonomy.

In our previous research (in press, D.M. Feda et al., *Journal of Science and Medicine in Sport*), when increasing choice from 1 toy to 3 or 5 active toys, boys played with more toys and increased their total physical activity time, yet decreased time playing with their favourite active toy by only 39%. Providing boys with one highly liked active toy is enough to motivate their physically active play. Boys' consistent time of play with their favourite toy effectively dampens the effect of choice on increasing active play time. In contrast, girls' reduced their play time with their most liked active toy 180% when given the choice to play with other active toys, so that choice becomes a salient factor increasing their active play. While the physical activity of many boys is below recommendations, the consistently greater physical activity of boys than girls across environments that vary in autonomy may limit the ability of some boys to increase their activity in environments specifically designed to increase autonomy. As such, autonomy-supportive environments may be less important for motivating boys' than girls' physical activity.

The girls in the present study reported lower enjoyment of physical activity and their enjoyment was positively correlated with their total activity during free-play of traditional active indoor games. Children experience enjoyment when they perform an activity that they are intrinsically motivated to engage in [4]. We did not specifically measure enjoyment of the choice and no-choice conditions, but providing autonomy through choice increased the motivation of the girls to be physically active as measured by an increase in physical activity and consequentially may have made the physical activity experience more enjoyable. If so, such repeated increases in situational motivation could promote increases in contextual motivation [44], which would encourage future decisions of girls to engage in active indoor play rather than being sedentary when at home.

We hypothesized that the mastery aspects designed into exergames would result in greater physical activity time when no choice was available. Indeed, children played exergames about twice as long as traditional games when only 1 game was available (Figure 1, bottom panel), suggesting that mastery motivated exergame play in the absence of an autonomy-supportive environment. Also, as predicted (Figure 1, bottom panel), an environment that provided both autonomy and mastery was most efficacious at increasing physical activity time. These results are consistent with those of Standage and colleagues [6] who found that both an autonomy-supportive environment and perceptions of a mastery climate were independent constructs positively associated with mediators (e.g., autonomy, competence, relatedness) of self-determined motivation. Self-determined motivation was then found to positively predict leisure-time physical activity intentions. Likewise, a mastery climate and satisfaction of the need for autonomy, competence, and relatedness were found to independently predict adolescent soccer players vitality for soccer [45].

Though mastery appears to help motivate children to play exergames twice as long as traditional games, given the differences in movement required to play exergames and traditional games, the energy expenditure of an exergame session may not be greater than traditional games. The current research extends the body of evidence regarding the energy expenditure of exergames by testing differences in the energy expenditure of exergames and traditional home indoor versions of the same games. Previous research has compared the cost of exergames to sedentary games or ergometer-type exercise such as treadmill walking or riding a cycle ergometer [20-28,46]. It is important to know the physical activity time and energy expenditure associated with exergames relative to traditional active toys in order to determine the implications of their use as an alternative to traditional active play. Though children in the current study played exergames twice as long as traditional games, this was off-set by the energy expenditure during play of exergames being approximately one-half that of traditional games. Thus, children must play exergames twice as long to match the energy they would have expended during traditional indoor play. Most of the exergames tested produced only small increases in energy expenditure above rest. Many exergames can be played using one arm and with little movement of the lower limbs. Exergame play requires movement of both upper limbs and the lower limbs to effectively increase energy expenditure [47,48]. An area for future study is to determine which types of activities exergame play replaces. If exergames replace traditional active play, that could result in a reduction in MVPA given that children participated in 142% greater MVPA when playing with traditional active toys than exergames. On the other hand, if exergame play displaces what would otherwise be sedentary videogame play or other sedentary behaviours, then even the light intensity physical activity of exergames would increase energy expenditure.

The basic laboratory data reported here add to the development of future intervention studies. The results suggest that incorporating aspects of both autonomy and mastery into a physical activity program may be best at increasing childrens' intrinsic motivation to participate in physical activity. Such a program may be most efficacious for girls, in particular, and may do so by making the exercise sessions more enjoyable.

This study is not without limitations. The children were tested alone. Typically play is a social behaviour, and children may have felt the isolated play environment was artificial. Children may have changed their total minutes of play, intensity of play or toy choices if they had been given more space or had a friend to play with. However, the traditional and exergames used to promote physical activity in the indoor laboratory setting were designed for indoor use and to be played either alone or with a peer or parent. Still, these are interesting questions for future research. The study was not powered to detect interaction effects of the energy expenditure data. Such results should be interpreted with some caution. Moreover, the small subsample size used to test differences in energy expenditure of exergames and traditional versions of the same games may limit generalizability.

## Conclusions

In conclusion, this study extends previous research of the basic factors that influence physical activity behaviour by showing that both autonomy and mastery increased intrinsic motivation for physical activity as measured by physical activity behaviour. However, an environment that provided both autonomy and mastery was most efficacious at increasing physical activity time. The effect of autonomy on increasing physical activity was greater in girls than boys. The present study, then, provides further evidence that boys and girls can differ in their behavioral responses to autonomy supportive environments. These gender differences in responsiveness to autonomy may stem from gender differences in usual physical activity levels and enjoyment of physical activity, and a lower need of choice for promoting physical activity of boys' due to their consistent time being active across different environments. Future research should further explore the mechanisms of the gender difference in responsiveness to autonomy-supportive environments. It is important to continue to conduct laboratory studies to identify basic factors that influence physical activity behaviour of girls and boys so that these factors can be translated to improve the efficacy of treatment programs that promote sustained increases in youth physical activity. Such work will provide important information for designing physical activity programs that children will be the most motivated to regularly participate in. Health care professionals, physical educators, and parents should understand that exergame play cannot be equally substituted for traditional active play. Children must play exergames twice as long to match the energy they would have expended during traditional indoor play. These individuals must also recognize that the energy expenditure during exergames and traditional active play outdoors would likely be much greater than the differences reported here between exergames and active play of traditional indoor games.

## Competing interests

The authors declare that they have no competing interests.

## Authors' contributions

JNR conceived of the study and its design, participated in its coordination and drafted the manuscript. MJL and TFM made substantial contributions to the coordination of the study, acquisition of data and helped to revise the manuscript. DMF assisted with the study design and helped to revise the manuscript. KFK lead the ergospirometry measurements and helped to revise the manuscript. All authors read and approved the final manuscript.
